# Risk Factors, Clinical Manifestations, and Outcomes of COVID-19-Associated Mucormycosis and Other Opportunistic Fungal Infections

**DOI:** 10.7759/cureus.46289

**Published:** 2023-09-30

**Authors:** Dinesh Kumar, Faiz Ahmad, Anil Kumar, Mamta Bishnoi, Anoop Grover, Parveen Rewri

**Affiliations:** 1 Otolaryngology, Maharaja Agrasen Medical College, Agroha, IND; 2 Otolaryngology, Head and Neck Surgery, Maharaja Agrasen Medical College, Agroha, IND; 3 Ophthalmology, Maharaja Agrasen Medical College, Agroha, IND; 4 Dentistry, Maharaja Agrasen Medical College, Agroha, IND

**Keywords:** mortality, immunosuppression, covid associated mucormycosis, opportunistic fungal infections, mucormycosis, covid-19

## Abstract

Introduction: An epidemic of opportunistic fungal infections during the second wave of the coronavirus disease 2019 (COVID-19) pandemic badly affected India in 2021. Several unknown, unique factors played a role in its causation and survival outcomes, including the severe acute respiratory syndrome coronavirus 2 (SARS-CoV-2) infection. The purpose of this study was to analyse the probable underlying risk factors and to know immediate and late outcomes of opportunistic fungal infections in the unique setting of the SARS-CoV-2 pandemic.

Methods: In this retrospective cohort study, clinical records of COVID-19-associated opportunistic fungal infections were reviewed for risk factors, clinical features, microbiological and pathological findings, and outcomes during a one-year follow-up at a tertiary care teaching hospital in Northern India.

Results: A total of 390 patients were admitted with symptoms and clinical signs consistent with the criteria for the diagnosis of COVID-19-associated mucormycosis (CAM). Diabetes mellitus was the most common comorbidity (74%). During the management of SARS-CoV-2, 192 (49%) patients received corticosteroids, 151 (39%) were on oxygen support, and 143 (37%) used at-home steam inhalation. Masks of any type were used by 236 (60.5%) patients, of whom most used cloth masks (n=147, 37.6%). Microbiologically, fungal growth was positive in 138 (35.3%) samples; of these, 74 (19%) had non-Mucorales fungal colonies. The fungal infection invaded structures beyond the paranasal sinuses in 60% of the cases. The overall mortality in this cohort after one-year follow-up was 40.25%.

Conclusions: An alignment of several predisposing conditions precipitated an epidemic of opportunistic fungal infections during the COVID-19 pandemic that resulted in high mortality in affected patients.

## Introduction

The world, and largely India, suffered an epidemic of mucormycosis during the second wave of the coronavirus disease 2019 (COVID-19) pandemic in 2021 [1]. The official figure for the total number of COVID-19-associated mucormycosis (CAM) cases in India was 51,775 till November 2021 [2]. Mucormycosis, colloquially known by the name ‘black fungus’ in Indian media and on social media platforms during COVID-19, was reported in the majority of cases, but many other non-Mucorales opportunistic fungi were also isolated [3,4]. Opportunistic fungal infections (or mycoses) mainly affect persons who have compromised immunity secondary to diabetes, malignancy, HIV, or the use of immunosuppressive drugs [5]. In India, uncontrolled diabetes mellitus is the single most important predisposition for opportunistic mycoses [6].

During the COVID-19 pandemic, an unusual alignment of multiple risk factors could have triggered an epidemic of opportunistic fungal infections. Systemic steroids were extensively used to treat moderate-to-severe cases of severe acute respiratory syndrome coronavirus 2 (SARS-CoV-2) infection, which caused hyperglycaemia and immunosuppression, both of which are known risk factors for opportunistic mycoses. In addition to these, some unique precautionary and therapeutic practices such as prolonged use of masks, steam inhalation, oxygen support, and immunomodulators (tocilizumab) were prevalent during COVID-19 [7]. Further, the management of CAM in India was affected by the shortage of anti-fungal liposomal amphotericin-B [8,9].

This study reviews demographics, risk factors, clinical presentations, concurrent mortality, and survival rates after one year of follow-up in a large cohort of Indian patients admitted with opportunistic fungal infections at a tertiary care hospital to know why opportunistic infections hit India at such a large scale, did mask use and steam inhalation contribute, and how the availability of anti-fungal drugs affected the outcomes of opportunistic mycoses in the backdrop of SARS-CoV-2.

## Materials and methods

The retrospective study was done in a tertiary care teaching institute in the Northern Indian state of Haryana. The study protocol was approved by the ethical committee of Maharaja Agrasen Medical College (MAMC/ICE/2022/58; dated 05.12.2022) and adhered to the tenets of the Declaration of Helsinki. 

Our hospital was designated a referral centre for the management of SARS-CoV-2, and later of CAM, for five districts of Haryana. For this study, we reviewed case records of all the patients admitted with a clinical diagnosis of CAM between 28th April and 10th December 2021. The diagnosis was based on clinical symptoms, endoscopy findings, radiological imaging (contrast-enhanced CT scan/MRI), direct microscopy (potassium hydroxide (KOH) wet mount), microbiological culture, and pathological evidence [10,11]. The inclusion criteria included patients suspected of having a fungal infection of the nasal cavity or paranasal sinuses, supported by the evidence used to establish the diagnosis. Patients with bacterial, other non-fungal infections, and non-infective sinusitis were excluded.

These patients underwent functional endoscopic paranasal sinus surgery (FESS) with debridement of necrosed tissue. The diseased tissue was sent for microbiological (KOH mount and culture) and/or histopathological confirmation. Additional concurrent or subsequent surgical interventions were decided on a patient-to-patient basis. This included partial or total maxillectomy, intra-orbital injections of amphotericin B, orbital exenteration, neurosurgical intervention, and laparotomy. 

After initial surgical intervention and baseline renal function tests (RFT), CAM patients were started on once daily, slow (over four to five hours) intravenous infusion of 5mg/kg/day liposomal amphotericin B or 1mg/kg/day amphotericin B conjugated with deoxycholate (AMBD), depending on the availability from the government. Periodic RFTs were done and treatment was withheld if any derangement of RFT occurred. The accumulative dose of 5g was infused. On completion of parenteral therapy with amphotericin B oral posaconazole was started for 90 days with intermittent follow-ups. Patients in which non-Mucorales fungi were isolated were switched to a loading dose of intravenous voriconazole (6mg/kg/day) followed by 4m/kg/day for five days and then 200mg/day oral. All stable patients on non-parenteral treatment were discharged and followed for one year (till December 2022). Based on the level of evidence, retrieved case records were categorised as possible, probable, and proven. The proven cases had definitive evidence of fungal infection either on direct microscopy, culture, or histopathology. Case records, in which isolated fungi belonged to order Mucorales, were labelled as rhino-orbital-cerebral mucormycosis (ROCM). If fungi belonged to non-Mucorales order, it was called opportunistic mycoses other than mucormycosis. Case records in which diagnosis was based on either endoscopy findings or radiological signs were categorised as probable ROCM, and those in which diagnosis was based solely on typical clinical symptoms and signs were categorised as possible ROCM (Table 1).

**Table 1:** The criteria used to categorise the patients of rhino-ocular-cerebral-mucormycosis (ROCM) based on strength of evidence Adopted with permission from Honavar SG, 2021 [10]

Category	Criteria
Possible ROCM	Clinical symptoms or signs
	Pain/swelling/numbness affecting face, cheeks, eyelids
	Discolouration of peri-orbital/ peri-oral skin/tongue/gums
	Ptosis/ diplopia/decreased vision/ protrusion of eyeball
	Black or blood-stained nasal discharge
Probable ROCM	Endoscopic or radiological evidence (contrast-enhanced CT/MRI)
	Blackening of middle turbinate
	Inflamed/congested nasal mucosa
	Blood-stained nasal discharge
	Purulent discharge in middle meatus Bone destruction and/or orbital infiltration
Proven ROCM	Microbiological or histopathological evidence
	Fungal element with hyphae on KOH wet mount
	Microbiological culture showing fungal elements with non-pigmented, no-/pauci-septate hyphae with variable width (6-16µm)
	Tissue biopsy with pathological evidence of tissue invasion, vessel occlusion

We recorded epidemiological data including demographics, risk factors, and clinical presentations as per the guidelines for outbreak investigation in India [12]. The information on risk factors included pre-existing or recently diagnosed diabetes mellitus, other co-morbid conditions, use of corticosteroids, the prevalent use of interventional and behavioural therapies such as oxygen support, steam inhalation, types, and usage pattern of masks. Similarly, information about microbiological, histopathological, and radiological investigations was retrieved and descriptive analysis was done. The clinical staging was done based on the extent of involvement. The mortality was calculated as concurrent and late. Death during hospital stay was defined as concurrent mortality and death after discharge was entered as late mortality.

The results were compiled using descriptive statistics and values were expressed as proportions, mean and medians.

## Results

**Figure 1:**
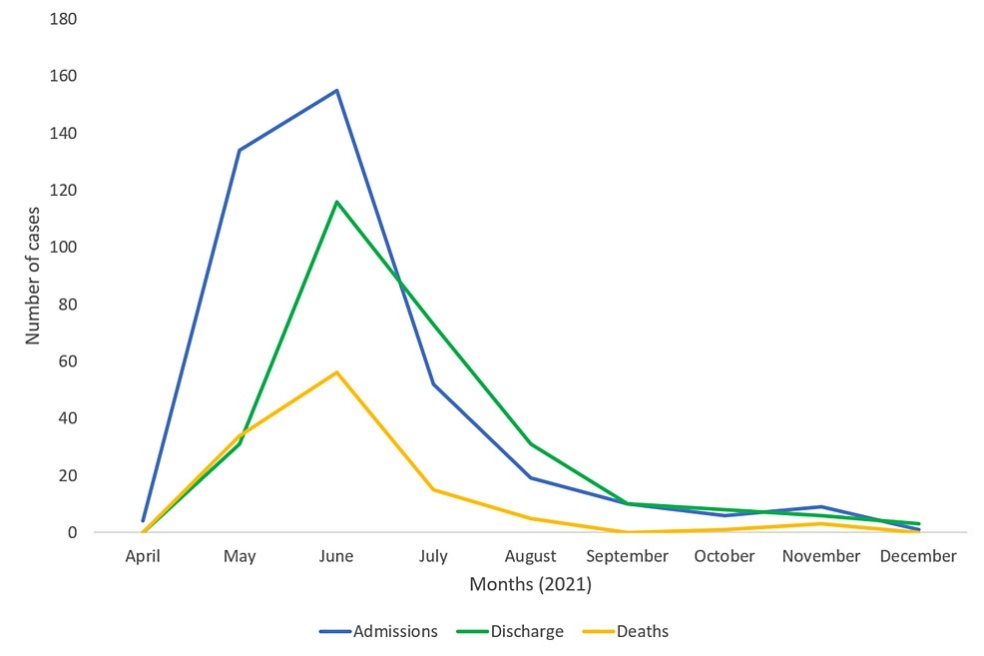
The epidemic trend curve showing admissions, survival, and mortality during study period

A total of 390 patients, 275 (70%) men and 115 (30%) women, were admitted during the selected study period. The mean ±SD (range) age of the cohort was 54±13 (14-88) years; 29% (n=114) of the patients were aged over 60 years. Vocational data was available for 324 (83%) patients, 232 (84% of 275) men, and 92 (80% of 115) women. Most of the men were farmers (n=106, 46%), and most of the women were homemakers (n=86, 75%). The majority (73%) belonged to rural areas. The peak was observed in June (Figure 1).

**Figure 2:**
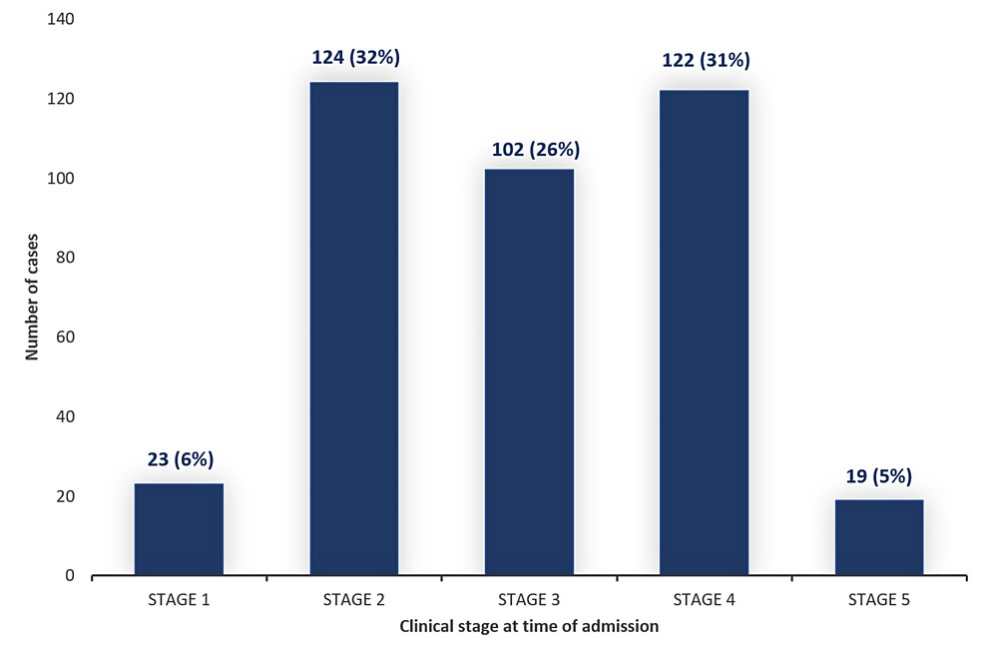
Histogram showing number and proportion of cases admitted at different clinical stages

**Figure 3:**
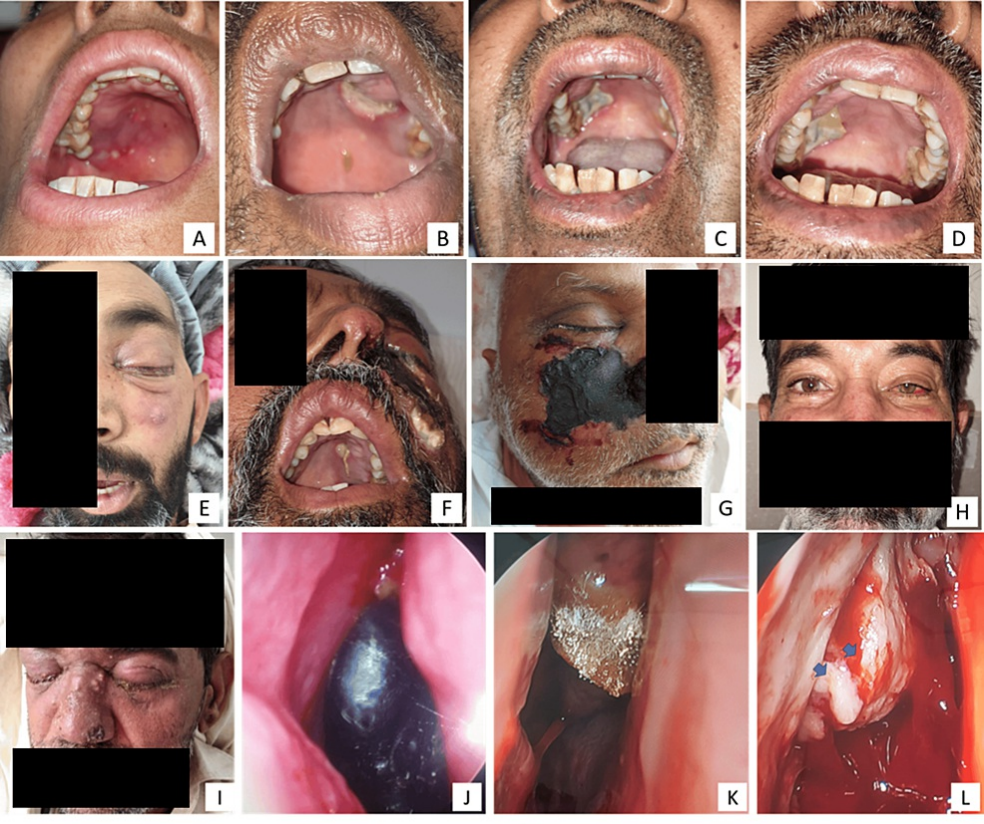
Representative cases of opportunistic fungal infections. (a) Multiple ulceration over the hard palate, a characteristic feature of mucormycosis, (b) A large necrotic patch over hard palate and alveolar bone on left side of maxilla, (c) Palatal ulceration and necrosis with sloughing of palatal mucosa, (d) Bilateral involvement - necrosis of right palatal and left alveolar bone, (e) Cutaneous ulceration on left anterior cheek through perforation in maxillary sinus and total ophthalmoplegia, (f) Palatal necrosis, sinus perforation over cheek skin and left eye proptosis, (g) Skin necrosis overlying maxillary sinus, total ophthalmoplegia with facial palsy on right side, (h) Left side ptosis and conjunctival congestion, (i) Bilateral orbital cellulitis, (j) Endoscopic view of right side middle turbinate necrosis, (k) Endoscopic view of fungal colonies on anterior middle turbinate, (l) Endoscopic view of pus oozing from right maxillary sinus after middle turbinate removal (blue arrowheads).

The rhino-paranasal (31.7%) and rhino-orbital-cerebral (31.2%) cases admitted to the hospital accounted for the largest proportion of cases (Figure 2), most at stage 4d (Table 2). Representative cases are shown in Figure 3.

**Table 2:** Distribution of all 390 cases, including non-Mucorales fungi, based on clinical stage Adopted with permission from Honavar SG, 2021 [10]

Clinical stage		Number (%)	
Stage	Structure affected/lesions		
Stage 1: Involvement of nasal mucosa			
1a	Middle turbinate	06	(1.5)
1b	Inferior turbinate/ostium of nasolacrimal duct	05	(1.5)
1c	Nasal septum	08	(2)
1d	Bilateral nasal mucosa	04	(1)
Stage 2: Involvement of paranasal sinuses			
2a	Ipsilateral single sinus	09	(2)
2b	Ipsilateral two sinuses	35	(9)
2c	Ipsilateral more than two sinuses/palate/oral cavity	24	(6)
2d	Bilateral paranasal sinuses/mandible/zygoma	56	(14)
Stage 3: Involvement of orbit			
3a	Nasolacrimal duct/medial orbit with vision unaffected	02	(0.5)
3b	Diffuse orbit involvement with vision unaffected	55	(14)
3c	Central retinal artery or ophthalmic artery occlusion/superior ophthalmic vein thrombosis/superior orbital fissure/inferior orbital fissure/orbital apex/vision affected	28	(7)
3d	Bilateral orbits	17	(4)
Stage 4: Involvement of central nervous system			
4a	Cribriform plate/incomplete cavernous sinus	00	
4b	Cavernous sinus thrombosis/diffuse involvement	07	(1.5)
4c	Internal carotid artery occlusion/ focal brain parenchyma infraction/skull base	01	(0.25)
4d	Multifocal/diffuse brain lesions	114	(30)
Stage 5: Systemic dissemination			
5	Lung/gastrointestinal tract/renal	19	(5)

**Figure 4:**
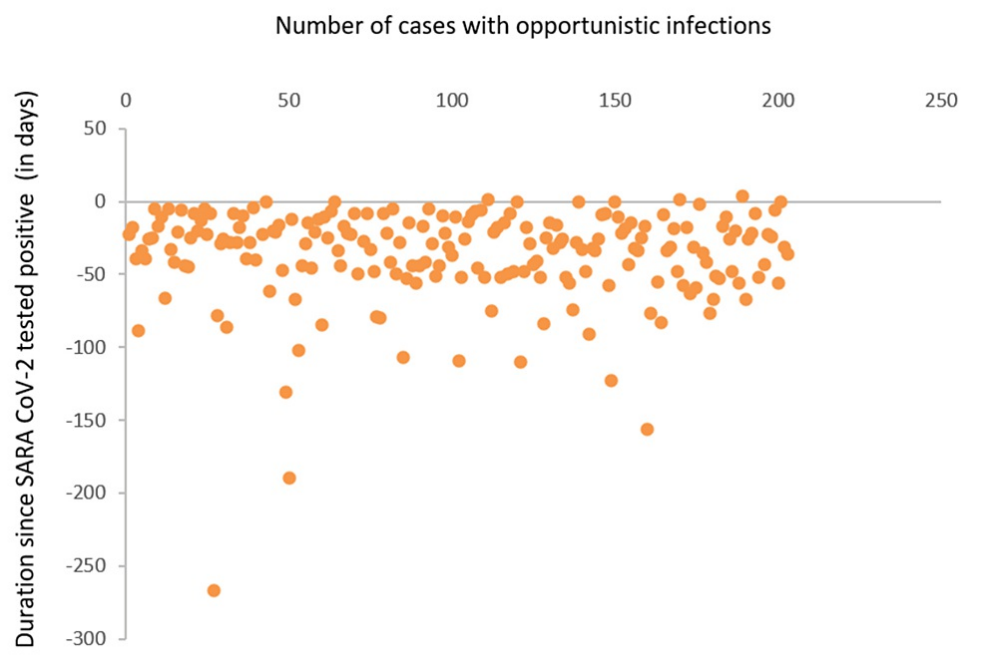
Scatter diagram depicting duration between severe acute respiratory syndrome coronavirus 2 (SARS-CoV-2) and opportunistic fungal infection

SARS-CoV-2 infection preceded the onset of symptoms suggestive of CAM in 203 (52%) patients with a median duration of 23 days (Figure 4). Fever history within the six-week period preceding the onset of symptoms was noted in 318 (82%) case records. Information about vaccination status against SARS-CoV-2 was available in 329 (84%) case records: 50 (12.8%) patients had received one dose, 11 (2.82%) had received two doses, and 268 (68.7%) had denied receiving any dose of vaccine.

The fungal infection was evident on KOH mount in 91 (20.7%) cases, on culture in 138 (35.3%), and on biopsy in 112 (28.7%) cases. After excluding 74 (19%) case records, in which fungi other than those of the order Mucorales were isolated on culture, a total of 316 case records were analysed for CAM. Of these, 150 (47%) were classified as proven ROCM, 101 (32%), as probable ROCM, and 65 (21%) as possible ROCM. The isolated fungi belonging to Mucorales order included those of genera Rhizopus, Mucor, Rhizomucor, and Phycomyces. Those belonging to non-Mucorales included those of genera Alternaria, Aspergillus, Candida, Curvularia, and Trichophyton. 

In the cohort of these 390 patients, 302 (77%) had co-morbidities, most commonly diabetes mellitus (294, 74%). The mean±SD random glucose level at the time of admission was 274±122 mg%. The other co-morbidities were seen in 56 (14%) patients, which included hypertension (n=38, 9.7%), chronic obstructive pulmonary disease (n=5, 1.2%), viral-hepatitis (n=12, 3%), malignancies (n=4, 1%), and HIV (n=2, 0.5%).

**Figure 5:**
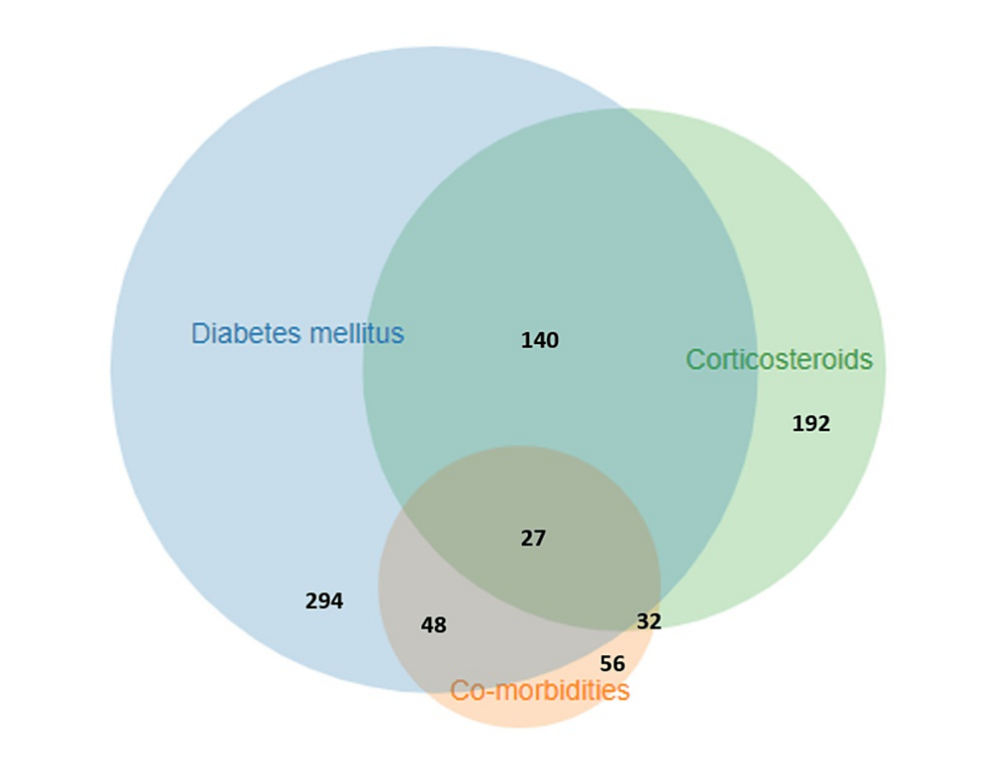
Venn diagram showing distribution of major risk factors

The documented use of corticosteroids during treatment for SARS-CoV-2 was available for 192 (49%) patients; in the remaining 198 (51%) cases, there was no clear evidence of steroid use. A total of 140 (47.6% of 294) diabetic patients received corticosteroids (Figure 5). Oxygen support was received by 151 (39%) patients during the SARS-CoV-2 infection. Steam inhalation was practiced by 143 (37%) patients; of these, 62 (16%) also received oxygen therapy. Use of any type of mask was practiced by 236 (60.5%) patients, of whom most used cloth masks (n=147, 37.6%), followed by surgical (n=63, 16%) and N-95 (n=27, 7%). The majority (n=174, 74% of 236) of the mask users changed or washed their mask(s) daily, whereas 26% (n=61) admitted reusing their mask more than a day. No mask was used by 93 (23.8%) patients.

**Figure 6:**
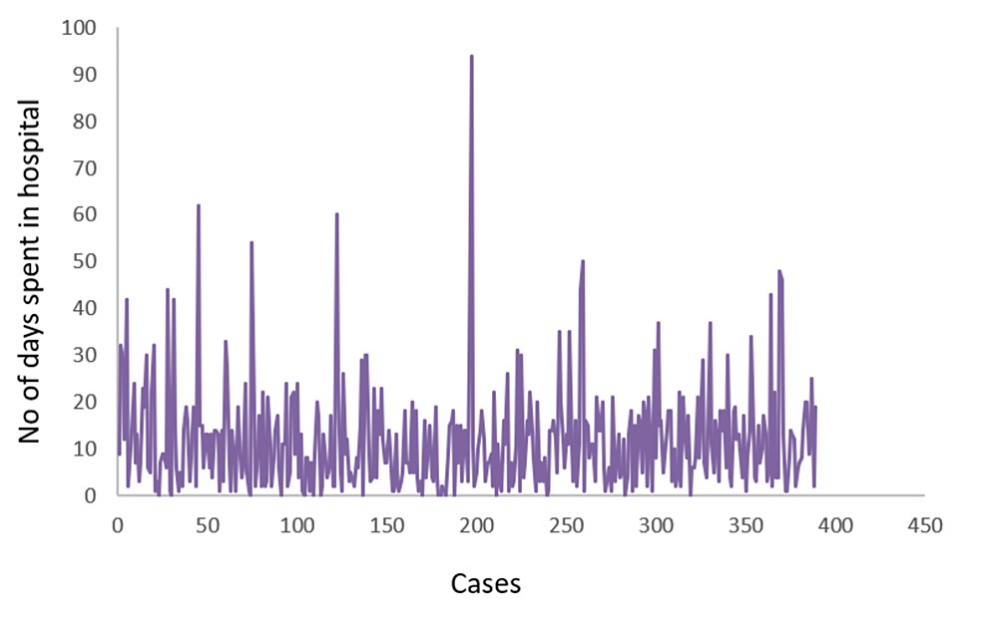
The duration of hospital stays until outcome (discharge or death)

FESS with surgical debridement was done in 353 (90%) patients. In 81 (21%) patients partial or total maxillectomy was done, and in nine (2%) patients orbital exenteration was carried out. One patient underwent craniotomy. The mean duration of amphotericin therapy was 9±8 days. The median duration of stay (till discharge or death) in the hospital was nine days (Figure 6). Recurrence was seen in 36 (9.2%) cases who were re-admitted for surgical debridement followed by standard management with pharmacotherapy. The overall mortality in this cohort at one year of follow-up was 40.25% (157 of 390), which included early mortality in 112 (28.7%) patients and late mortality in 45 (11.5%) patients. Mortality among those who underwent additional surgical procedures (maxillectomy or orbital exenteration) was 22%. In proven cases, mortality was higher in patients with non-Mucorales (n=27; 37% of 74) infection compared to those infected with Mucorales (n=45; 30% of 150).

## Discussion

Mucormycosis among SARS-CoV-2 patients was reported from different parts of the world during the second wave of the COVID-19 pandemic; India alone contributed over 96% of cases [4]. The reasons for the CAM epidemic in India are not entirely clear. We believe this was because of the high prevalence of mucormycosis in India (140 cases per million population), which is 70 times higher than the global data [13,14]. The high prevalence is attributed to uncontrolled diabetes, a tropical or sub-tropical humid climate, and high environmental temperatures in most parts of India [13,14]. The rate of fungal infections is also affected by demographic and socio-economic factors and is more common among men and rural inhabitants [15].

The COVID-19 pandemic precipitated the mycoses epidemic in predisposed populations when conducive weather conditions favored fungal growth. In our study, we found that nearly three-fourths of our patients were from rural backgrounds, and nearly 46% of men were farmers. Approximately 80% of cases were reported mainly between May and July 2021, and a peak was observed in June. The weather during this duration was humid with record high temperatures [16]. In our study, nearly two-thirds of patients had hyperglycaemia, and nearly 50% used corticosteroids.

The SARS-CoV-2 infection affects host immunity [17]. Did SARS-CoV-2 cause an immunocompromised state itself and predispose to opportunistic fungal infections? This can be answered only through a case-control study. Indirect evidence that SARS-CoV-2 infection led to an immunocompromised state was that 11% of patients in this study had no known risk factor, i.e., diabetes mellitus, corticosteroid use, any other co-morbidity, or neutropenia. The hosts of mucormycosis infection are immunocompromised in 80% of cases [14,18]. Important risk factors for mucormycosis are diabetes mellitus, corticosteroids, and neutropenia [11].

We presume that some of the preventive practices observed by the common man during the COVID-19 pandemic provided a moist local environment favoring oral/nasal fungal growth. Oxygen support in hospitalised patients, steam inhalation by mild non-hospitalised patients or as a preventive measure, and prolonged mask use were common during the COVID-19 pandemic [7,19,20]. At-home steam therapy was endorsed as a preventive measure against SARS-CoV-2 infection in India [7].

Arora et al. found that the duration of mask use, irrespective of its type (surgical or cloth-made), was associated with the risk of CAM when compared to the control group of COVID-19 patients [20]. However, cloth mask use, compared to surgical mask use, for even less than two hours predisposed users to CAM [20]. Moisture retention, reuse, and poor filtration increased the risk of infections with cloth masks [21]. The lay media attributed the CAM epidemic to industrial oxygen when, due to lack of medical-grade oxygen supply, industrial oxygen was diverted to hospitals [22]. Though it would be hard to prove what role each individual predisposition factor played, it is understandable that the exponential rise of opportunistic infections was the result of some of these previously unappreciated risk factors, including SARS-CoV-2 itself.

Despite similarities in clinical presentation, in 19% of cases, non-Mucorales opportunistic fungi were isolated. Microbiological differentiation is important as the clinical course and treatment are different. Mucormycosis primarily affects the sinuses and can spread to the eyes, brain, and other organs. In our study, we found that on presentation, infection had reached beyond the paranasal sinuses in more than 50% of cases.

Early mortality in our study was close to 28%, and an additional mortality of 14% was recorded during the one-year follow-up period. In these late deaths, exact cause of mortality could not be established, but it can be fairly assumed that deaths in these cases resulted from complications of SARS-CoV-2 or opportunistic infections. The overall mortality rate in our cohort was 40%. The reported mortality in mucormycosis ranges between 40% and 80%, depending on underlying conditions and sites of infection [11]. This would require more studies on how an epidemic marred by a shortage of liposomal amphotericin B affected survival outcomes. Demand and supply were badly mismatched in India due to a shortage of amphotericin B [8,9]. Increased mortality is associated with delayed initiation of therapy in mucormycosis [23]. We too suffered delays and irregularities in treatment regimens due to a shortage and inconstant supply of amphotericin B, but the overall mortality did not exceed that reported in pre-COVID-19 times.

There are some limitations to this study. Since it is a descriptive cohort, the precise role of risk factors for CAM or opportunistic mycosis could not be evaluated. We also could not study the impact of the irregular availability of amphotericin B on morbidity and mortality. Also, we could not find the cause of late mortality as this information was received on the phone after the patient did not turn up for follow-up.

## Conclusions

In conclusion, the SARS-CoV-2 infection and its management and environmental conditions favoured the growth of opportunistic fungal infections among predisposed people. It is imperative to establish diagnosis based on imaging, microbiology, and histopathological evidences because the non-mucorales order infections had clinical presentations like CAM. The role played by each individual factor would require controlled studies. At present, it seems appropriate to presume that high temperatures and humid conditions promoted the growth of fungi. The important lesson that must be learned from this epidemic is to judiciously use drugs and practices that may compromise the immune status of an individual. 
